# The Influence of In Vitro Gastrointestinal Digestion on the Anticancer Activity of Manuka Honey

**DOI:** 10.3390/antiox9010064

**Published:** 2020-01-10

**Authors:** Danila Cianciosi, Tamara Yuliett Forbes-Hernández, Sadia Afrin, Massimiliano Gasparrini, José L. Quiles, Emilio Gil, Stefano Bompadre, Jesus Simal-Gandara, Maurizio Battino, Francesca Giampieri

**Affiliations:** 1 Dipartimento di Scienze Cliniche Specialistiche e Odontostomtologiche, Università Politecnica delle Marche, Via Ranieri 65, 60130 Ancona, Italy; d.cianciosi@pm.univpm.it; 2 Nutrition and Food Science Group, Department of Analytical and Food Chemistry, CITACA, CACTI, University of Vigo, Vigo Campus, 32004 Ourense, Spain; tforbes@uvigo.es or; 3 Department of Gynecology and Obstetrics, Johns Hopkins University School of Medicine, Baltimore, MD 21205, USA; safrin1@jhmi.edu; 4 Department of Agricultural, Food and Environmental Sciences, Università Politecnica delle Marche, Via Brecce Bianche 10, 60131 Ancona, Italy; m.gasparrini@univpm.it; 5 Department of Physiology, Institute of Nutrition and Food Technology “Jose Mataix”, Biomedical Research Center, University of Granada, 18000 Granada, Spain; jlquiles@ugr.es; 6 College of Food Science and Technology, Northwest University, Xi’an 710069, China; 7 Nutrition and Food Science Group, Department of Biochemistry, Genetics and Immunology, Faculty of Biology, University of Vigo, 36310 Vigo, Spain; egil@uvigo.es; 8 Department of Biomedical Sciences, Polytechnic University of Marche, 60131 Ancona, Italy; s.bompadre@univpm.it; 9 Nutrition and Bromatology Group, Department of Analytical and Food Chemistry, CITACA, Faculty of Science, University of Vigo, Ourense Campus, E-32004 Ourense, Spain; jsimal@uvigo.es; 10 International Research Center for Food Nutrition and Safety, Jiangsu University, Zhenjiang 212013, China

**Keywords:** bioaccessibility, bioavailability, colon cancer, honey, polyphenols, in vitro simulated digestion

## Abstract

Manuka honey (MH) is a natural food with many beneficial properties to human health, thanks to its high variety of bioactive compounds; however, little is known about its bioaccessibility. The aim of this study was to evaluate and compare the polyphenol compounds, the antioxidant capacity and the anticancer activity of MH subjected to an in vitro gastrointestinal digestion in human HCT-116 colon cancer cells. Raw MH and digested MH (DMH) were assessed for total polyphenols and flavonoids by spectrophotometric and HPLC-ESI-MS/MS analysis, and total antioxidant capacity (TAC) using different methods. Cell viability, intracellular ROS production, apoptosis, cell cycle and colony formation capacity were tested after treatment with MH or DMH. Results showed that total polyphenols, total flavonoids and TAC were significantly (*p* < 0.05) reduced after in vitro digestion. In addition, MH and DMH at 8, 16 and 24 mg/mL had similar effects in inducing intracellular ROS production and in inhibiting the colon formation ability; MH induced a more marked apoptosis compared to DMH, while cell cycle was blocked in S phase by MH and in Sub G1 phase by DMH. Our results increase knowledge of the effect of gastrointestinal digestion on the biological effect of honey against colorectal cancer.

## 1. Introduction

Colorectal cancer is one of the three most common types of cancer diagnosed in the world and its survival rate is also very low; it is, in fact, in second place for mortality. Genetic and environmental factors are mainly responsible for the onset of this type of tumor. Among the environmental factors, in addition to alcohol consumption and smoking habits, diet plays a relevant role: in fact, it has been confirmed that in obese and/or diabetic subjects the risk of colon cancer onset is much higher and this is associated with high levels of insulin, estrogens, and insulin-like growth factor (IGF-1). The consumption of red or processed meat has also been shown to be associated with a higher risk of onset, due to a greater presence of heterocyclic aromatic amine, *N*-nitroso compounds, N-alkyl amines and heme iron. On the contrary, a positive association with fiber consumption, fish rich in omega 3-fatty acids and Vitamin D was noted [1]. An even more evident positive association is with the consumption of fruit and vegetables, whose beneficial effect is exerted by the numerous vitamins and polyphenols that are able to modulate different pathways, many of which are associated with antioxidant and anti-inflammatory effects [2]. A natural substance of vegetable origin with these proven activities is honey, used since ancient times for the treatment of wounds and burns, gastrointestinal disorders and respiratory infections [3]. The evaluation of the effect of honey on various pathologies has been extensively studied in vitro and its antimicrobial, antidiabetic potential, its protective effect against the cardiovascular, respiratory and gastrointestinal system has been confirmed, as well its anti-proliferative effect against cells derived from different types of cancer, alone or in association with the chemotherapeutic agent commonly used to counteract these neoplasms, finding, in most cases, a synergistic effect [4]. On the other hand, the scientific literature on how the digestion process affects and modifies the bioavailability and bioaccessibility of the compounds responsible for this activity, particularly polyphenols, is scarce.

The bioavailability of polyphenols is a process that involves several factors, not only related to the food matrix—in fact, the interaction with other compounds, such as sugars and proteins, the structure of the single polyphenolic compound, the composition of the subject’s microflora and some other external factors must be taken into consideration [5]. Bioaccesibility, on the other hand, is a factor that depends strictly on the type of food matrix and the digestion process; in fact, the definition of a bioaccessible portion is “the fraction of compounds and/or nutrients that is released during the gastric-intestinal process from the food matrix and that is available for subsequent absorption” [6].

In vitro simulation of gastrointestinal digestion can be used to study the bioaccesibility of bioactive compounds from the food matrix. This method is rapid, economic, safe and does not have ethical restrictions [7].

The few studies present in the literature are limited to assessments of the polyphenol content and the antioxidant capacity of different types of honey (manuka, multifloral, bracatinga), subjected to the in vitro, simulated gastric-intestinal digestion process, and they rarely evaluated if this process could “modify” the biological effect shown in cell lines. In this work, how the total polyphenolic content and the antioxidant capacity of Manuka honey (MH) (*Leptospermum scoparium*), both raw and subjected to the process of gastric-intestinal digestion in vitro, (DMH) changes, was initially evaluated. Subsequently, the biological effect (ROS production, apoptosis rate, effect on cell cycle and colony formation) of MH and DMH on colon-rectal adenocarcinoma cells (HCT-116) was evaluated.

## 2. Materials and Methods

### 2.1. Honey Sample and Reagents

Manuka HoneyNèctar Plus^®^ certified pure and raw honey UMF 15+ (MGO 514) was imported to Italy by Efit S.r.l. from Timaru, New Zealand and kept at 4 °C until analysis. Folin and Ciocalteu’s phenol reagent, bile extract porcine, pepsin, pancreatin, glacial acetic acid (CH_3_CO_2_H), methanol (CH_3_OH), sodium carbonate (Na_2_CO_3_), sodium nitrite (NaNO_2_), aluminum chloride hexahydrate (AlCl_3_*6H_2_O), potassium peroxodisulfate (K2S2O8), sodium acetate (CH3COONa), ABTS™ (2,2′-Azino-bis(3-ethylbenzothiazoline-6-sulfonic acid) diammonium salt), 2,4,6-Tris(2-pyridyl)-s-triazine (TPTZ), 2,2-Diphenyl-1-picrylhydrazyl (DPPH) and ammonium iron(II) sulfate hexahydrate (H₈FeN₂O₈S₂·6H₂O) were purchased from Sigma Aldrich Chemie GmbH (Steinheim, Germany). (+)-catechin, ethanol (EtOH), (±)-6-Hydroxy-2,5,7,8-tetramethylchromane-2-carboxylic acid (TROLOX) and 3,4,5-trihydroxybenzoic acid (Gallic acid) were purchased from Fluka Chemie (Buchs, Switzerland). McCoy’s 5A and other reagents for cell culture were purchased from Corning^®^ (New York, NY, USA), and Sigma Aldrich (St. Louis, MO, USA) Tali Apoptosis assay™and Cell Cycle kit was bought from Invitrogen, LifeTechnologies.

### 2.2. Cell Culture

Human colon adenocarcinoma (HCT-116) cell line was purchased from the American Type Culture Collection (ATCC, Manassas, VA, USA). McCoy’s 5A media was used for HCT-116 cell culture; the media was supplemented with 10% heat-inactivated fetal bovine serum and penicillin/streptomicin 100 IU mL^−1^. The cell line was maintained in the incubator at 37 °C, 5% CO_2_. For all analyses, cells were used between the 4th and 10th passages.

### 2.3. In Vitro Digestion of Manuka Honey and Sample Preparation

**Figure 1:**
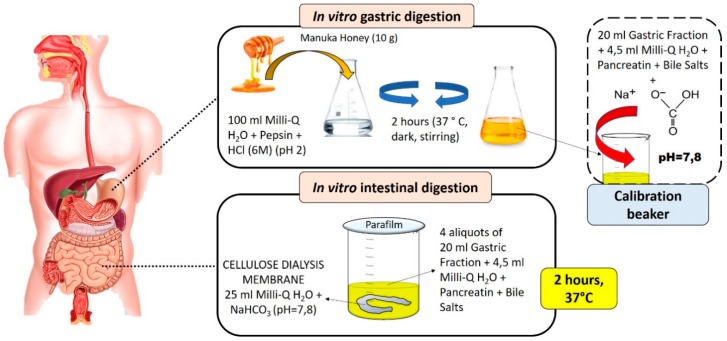
Graphical and schematic representation of the in vitro gastrointestinal digestion process.

The in vitro digestion of MH was performed following the method described by Gil-Izquierdo et al. [8], with some modification (Figure 1). Ten grams of MH were added to 100 mL of pure MilliQ water and, through the addition of HCl (6M), a pH of 2 is reached to simulate the gastric acid environment. To this solution, pepsin deriving from the porcine gastric mucosa (315 U/mL) was added in an amount of 0.734 mg/g of MH. The solution was incubated with agitation at 37 °C in the dark for 2 h. At the end of this incubation time, 20 mL of resulting gastric fraction were taken for the titration of the other aliquots, and added to 4.5 mL of distilled water with dissolved porcine bile salts (25 mg/mL) and pancreatin deriving from porcine pancreas (4 mg/mL). The necessary amount of sodium bicarbonate (NaHCO_3_) was added to this solution to reach a pH similar to that of the intestinal tract (pH = 7.8), and the amount added was noted. For the subsequent intestinal phase, the remaining 80 mL from the gastric fraction were divided into four beakers and 4.5 mL of mineral water containing pancreatin and bile salts was added to each one. Within four dialysis membranes of 20 cm consisting of cellulose (cut of 12,000 Da), 25 mL of distilled water were added to each one with the amount of NaHCO_3_ needed to reach intestinal pH. The dialysis membrane was closed with a knot and immersed in the glasses containing the gastric solution—bile salts and pancreatin—then, every beaker was closed with the parafilm and incubated for 2 h in water bath at 37 °C; at the end of this incubation phase, the content inside the dialysis membrane (bioaccessibile fraction) was recovered and used for subsequent analysis. This sample was then purified by centrifugation at 10,000 rpm at 4 °C for 10 min and, at the end, the sample was concentrated under vacuum at 37 °C; from the initial 10 g of fresh honey, after evaporation, we obtained 1.479 g of dried samples. For all spectrophotometric analysis, raw and digested MH were dissolved in 2 mL of MilliQ water and 0.5 mL of methanol (MeOH). In vitro gastrointestinal digestion was also carried out for distilled water and then, in all the spectrophotometric calculations, the water value was subtracted because the enzymes used in this process can interfere with spectrophotometric analyses.

### 2.4. Quantification and Identification of Phenolic Compounds of Manuka Honey and Digested Manuka Honey

#### 2.4.1. Estimation of Total Phenolic Content

Total phenolic content (TPC) was determined using the Folin–Ciocalteau method [9]. The samples were appropriately diluted to be within the standard calibration curve (0.003–0.15 mg/mL of Gallic acid (GA)). A total of 1250 μL of Folin–Ciocalteau reagent (10%) was added to 250 μL of sample or standard; the solution obtained was incubated in the dark for 5 min and then 1 mL of sodium carbonate (7M) (Na_2_CO_3_) was added. The resulting solution was left in the dark at room temperature for 2 h, at the end of which the absorbance was measured at 760 nm with the UV-Vis spectrophotometer (model DU^®^ 6400 Spectrophotometer, Beckman, Fullerton, CA, USA). The TPC was expressed in mg GAEq/g honey.

#### 2.4.2. Estimation of Total Flavonoid Content

The aluminium chloride method described by Ariza et al. [10] was used to determine the total flavonoid content (TFC). The samples were appropriately diluted to be within the standard calibration curve (0.0008–0.05 mg/mL of Catechin (Cat)). A total of 625 μL of distilled pure water and 37.5 μL of Sodium Nitrite (NaNO_2_) (5%) were added to 125 μL of standards or samples. Six minutes after the incubation of this solution, 75 μL of AlCl_3_ hexahydrate (10%) were added to the solution, which was left for 5 min to incubate. After this time, 250 μL of Sodium Hydroxide (NaOH) (4%) and 137.5 μL of distilled water were added at the solution. The absorbance was measured at 510 nm with the UV-Vis spectrophotometer (model DU^®^ 6400 Spectrophotometer, Beckman, Fullerton, CA, USA). The TFC was expressed in mg CatEq/kg honey.

#### 2.4.3. Quantification of the Total Antioxidant Capacity

For the quantification of the total antioxidant capacity (TAC), three different techniques were used: the ferric ion-reducing antioxidant power (FRAP) assay, the Trolox equivalent antioxidant capacity (TEAC) assay and the 2,2-diphenyl-1-picrylhydrazyl (DPPH) assay. The samples were appropriately diluted to be within the standard calibration curve and the dilution factor used was considered for the calculations.

##### FRAP Assay

The method of Deighton et al. [11] was used to determine the FRAP. For the construction of the standard curve (50–500 μM) the Ferrous ammoniuym sulphate·6H_2_O was used. A total of 150 μL of samples or standards was added to 1350 μL of FRAP solution. The FRAP solution was composed by 2,4,6-Tri(2-pyridyl)-s-triazine (TPTZ) (10 mM), Ferric chloride FeCl_3_·6H_2_O and Sodium acetate CH_3_COONa·3H_2_O (300 mM) in a ratio of 1:1:10. The solution was mixed with vortex and incubated at room temperature for 4 min. The absorbance was measured at 593 nm with the UV-Vis spectrophotometer (model DU^®^ 6400 Spectrophotometer, Beckman, Fullerton, CA, USA). The results were expressed in μmol FEq/100 g Honey.

##### TEAC Assay

The method of Re et al. [12] was used to evaluate the antioxidant capacity throughout the TEAC assay. For the construction of the standard curve (50–1000 μM) Trolox (T) was used. The ABTS solution (radical solution) was prepared by mixing ABTS (7 mM) and K_2_S_2_O_8_ (2.45 mM) in Milli-Q water for 12–16 h before carrying out the analysis, and the mix was stored at 4 °C in the dark. After this, 115 μL of the radical solution were dissolved in 10 mL of pure EtOH. A total of 1000 μL of this final solution was mixed with 10 μL of standards, samples or Milli-Q water (for the control), and after vortex for 20 s, the mixture was incubated for 90 s and the absorbance was measured at 734 nm with the UV-Vis spectrophotometer (model DU^®^ 6400 Spectrophotometer, Beckman, Fullerton, CA, USA). The results were expressed in μmol TEq/100 g Honey.

##### DPPH Assay

The method of Kumaran et al. [13] was used to evaluate the antioxidant capacity, evaluating the capacity to scavenge DPPH radical. For the construction of the standard curve (50–1000 μM) Trolox (T) was used. In the solution consisting of DPPH, methanol (400 μL) and 550 μL of EtOH (70%), 50 μL of samples, standards or Milli-Q water (for the control) were added. After 15 min of incubation in the dark at room temperature, the absorbance was measured at 515 nm with the UV-Vis spectrophotometer (model DU^®^ 6400 Spectrophotometer, Beckman, Fullerton, CA, USA). The results were expressed in μmol TEq/100 g Honey.

### 2.5. Individual Phenolic Compounds by HPLC-ESI-MS/MS

The determination of phenolic compounds was performed according to Seraglio et al. [14] using a Liquid Chromatography system (Agilent model 1260 Infinity, Palo Alto, CA, USA) coupled with triple quadrupole tandem mass spectrometry MS/MS AB SCIEX Triple Quad 3500 (AB Sciex, Foster City, CA, USA), equipped with an electrospray ionization source (ESI). The validation parameters, including detection and quantification limits and levels and linear range of the calibration curves, are in accordance with Seraglio et al. [14].

The chromatographic separation was achieved in a Phenomenex Luna C_18_ column (150 × 2 mm; 3 μm particle diameter). The flow rate adopted was 300 μL min^−1^, the injection volume was 5 μL, and the mobile phase was composed of Solvent A (water with 0.1% formic acid) and Solvent B (acetonitrile with 0.1% formic acid). The mobile phase gradient was programmed as follows: 98% A (*v*/*v*) from 0 to 4.0 min, 80–98% A (*v*/*v*) from 4.0–7.0 min, 10–80% A (*v*/*v*) from 7.0–14.0 min, 10% A (*v*/*v*) from 14.0–15.0 min, 10–98% A (*v*/*v*) from 15.0–17.0 min. The diverter valve was open during the first 1.9 min of the chromatographic run. For the mass spectrometric analysis, a turbo V™ source, operating in positive and negative ionization modes, was set with the following parameters: ion spray (IS) voltage: 4500 V; curtain gas: 25 psi; nebulizer gas (GS1) and auxiliary gas (GS2): 55 psi; source temperature: 400 °C. Nitrogen was used as the nebulizer and collision gas. The acquisition was performed in multiple reaction monitoring (MRM) mode, and the Analyst 1.6.2 software (AB Sciex, Foster City, CA, USA) was used for data acquisition and processing. The concentration of the individual phenolic compound was expressed in μg/kg honey.

### 2.6. Cell Viability Assay

HCT-116 cell lines were seeded in the 96-well plates at a density of 5 × 10^3^ cells/well in 200 μL medium. The cells were then left to adhere to the surface of the wells in the incubator at 37 °C overnight, and the next day the cells, treated with a concentration range of MH and Digested Manuka Honey (DMH), were dissolved directly in a complete media in a concentration range of 0–26 mg/mL for times of 24, 48 and 72 h. After the incubation time, the 3-(4,5-dimethylthiazol-2-yl)-2,5-diphenyltetrazolium bromide (MTT) was dissolved in RPMI medium and 30 μL was added per well, and the cells were then incubated for about 2 h and 30 min. At the end of this time, 100 μL of dimethyl sulfoxide (DMSO) was added to each well to dissolve the formazan crystals formed and the absorbance of the samples was measured at 590 nm with a microplate spectrophotometer (Thermo Scientific Multiskan EX, Monza, Italy). The percentage of viable cells was calculated with the sequent formula

$$\%\;viable\;cells=\frac{Abs_{treated\;cells}}{Abs_{untreated\;cells}}\times 100$$

### 2.7. Determination of Intracellular ROS Levels Throught the Tali^®^ Image-Based Cytometer

The cells were seeded in a 6-well plates at a density of 1.5 × 10^5^ cells/well in an amount of 2000 μL of medium, and the next day they were treated with varying concentrations of MH and DMH—0, 8, 16 and 24 mg/mL, dissolved in complete media—for 48 h. After this time, the cells were trypsinized and centrifuged at 1500 rpm for 10 min. After the centrifuge, the resulting pellet was resuspended in 500 μL of complete medium, to which 1 μL of CellROX^®^ (Monza, Italy) Orange Reagent was added. After an incubation period of 30 min at 37 °C, the samples were again centrifuged, the supernatant was removed and the pellet was resuspended in 100 μL of PBS. The samples were then analyzed with the Tali^®^ Image-based cytometer (Thermo Fisher Scientific, Milan, Italy), and the results were expressed as fold increase accumulation of ROS, with respect to the control.

### 2.8. Determination of Apoptotic Cells Throught the Tali^®^ Image-Based Cytometer

The cells were seeded in 6-well plates at a density of 1.5 × 10^5^ cells/well, in 2000 μL of medium, and the next day they were treated with varying concentrations of MH and DMH (0, 8, 16 and 24 mg/mL dissolved in complete media) for 48 h. After this time, the cells were trypsinized and centrifuged at 1500 rpm for 5 min and the resulting pellet was resuspended with 100 μL of Annexin V Binding Buffer (ABB), and 5 μL of Annexin V Alexa Fluor^®^488 (Monza, Italy) was added at this suspension. Then, it was centrifuged again for 10 min, after 20 min of incubation in the dark. The pellet was resuspended in 100 μL of ABB, and 1 μL of propidium iodide (PI) was added, and this solution was incubated at room temperature in the dark for 5 min. The samples were then analyzed with the Tali^®^ Image-based cytometer (Thermo Fisher Scientific, Milan, Italy), that evaluated the percentage of live, apoptotic and dead cells. The viable cells were Annexin V negative/PI negative; the apoptotic ones were Annexin V positive/PI negative, whereas Annexin/PI positive cells were identified by the instrument as dead cells. The results were expressed as fold increase with respect to the control.

### 2.9. Cell Cycle Analysis Throught the Tali^®^ Image-Based Cytometer

The cells were seeded in the 6-well plates at a density of 3.8 × 10^5^ cells/well in 2000 μL of medium, and the next day they were treated with varing concentrations of MH an DMH (0, 8, 16 and 24 mg/mL dissolved in complete media) for 48 h. After this time, the cells were trypsinized and centrifuged twice at 500 g for 5 min, the second one suspending the pellet in 500 μL of PBS. The cells were fixed with 500 μL of cold EtOH (70%) maintaining the cells in ice; the samples were then incubated at −20 °C overnight. After this time, the cells were washed with PBS and resuspended with 500 μL of staining solution, comprised of PBS, 0.1% Triton^®^ X, 20 μg mL^−1^ of PI and 0.2 mg mL^−1^ RNase A (Invitrogen). The samples were incubated for 30 min in the dark, at room temperature; they were then analysed with the Tali^®^ Image-based cytometer (Thermo Fisher Scientific, Milan, Italy). The results were expressed as the percentage of cells in each phase of cell cycle (Sub G_1_, G_0_/G_1_, S, G_2_/M) and all data were reported as the mean value of three independent analyses ± SD.

### 2.10. Colony Formation Assay

The method of Waghela et al. [15] was used to perform the colony formation assay with some modifications. The cells were seeded in the 6-well plates at a density of 5 × 10^5^ cells/well in 2000 μL of medium, and the subsequent day they were treated with varying concentrations of MH and DMH (0, 8, 16, 24 mg/mL dissolved in complete media) for 48 h. After this time the cells were trypsinized and they were re-seeded in a 6-well plate at a density of 1000 cells/well and they were left to grow for 12 days, when small colonies began to form. The colonies were fixed with methanol and stained with 0.2% methylene blue. The plating efficiency (PE) is the ability of a single cell to survive and to grow in the form of colony. The PE was defined by the following formula

$$\%\;PE=\frac{Number\;of\;colonies\;formed}{Number\;of\;cells\;seeded}\times 100$$

### 2.11. Statistical Analysis

The data were reported as a mean value of at least three independent analyses ± SD and the statistical analysis was assed using STATISTICA software (Statsoft Inc., Tulsa, OK, USA). The normality was calculated by the Kolmogorov-Smirnov test and the homogeneity of variance by the Levene test. For the significant differences, letters were acquired using one-way analysis of variance (ANOVA) followed by Tukey’s honest significant difference post hoc test (*p* < 0.05).

## 3. Results and Discussion

### 3.1. Quantification and Identification of Phenolic Compounds of Manuka Honey and Digested Manuka Honey

#### 3.1.1. Total Phenolic Compounds and Antioxidant Activity

Spectrophotometric analyses were performed to evaluate and compare the TPC, the TFC and the TAC of the MH and DMH. The results obtained are shown in Table 1.

As shown in the table, the TPC of MH was 1.27 mg GAEq/g honey, until it decreased about 6-fold in the DMH (0.203 mg GAEq/g honey). Similar results were found by O’Sullivan et al. [16], who monitored how the total content of polyphenols in different types of commercial honey changed, finding that, of the four types observed, even if Manuka honey had greatest amount of phenolic compounds, after in vitro gastrointestinal, it lost the greatest amount of phenolic compounds. Regarding the effects of gastrointestinal digestion on honey, there are no further studies, but, in several other food matrices, a significative decrease in the phenolic compounds with respect to the undigested sample was noted. For example, in the seeds of white grapes, the decrease in the value of the TPC was almost three times compared to the undigested seeds, and the trend was more or less similar in the pomace faction [17]. A clear decrease in TPC after gastrointestinal digestion was also observed in extracts of pomegranate [18], papaya, jackfruit [19], and in fresh persimmons [20]. However, there are some studies in which there are no clear differences between the TPC of the undigested and digested samples [16,21]. The decrease observed in the various studies, including this work, could be due to the fact that some phenolic compounds are more sensitive than others to pH changes, and moreover the type of food matrix could change their exposure to different conditions of acidity/basicity during gastro-intestinal digestion.

In contrast with the obtained results in this study, in other works an increase in TPC was observed after the in vitro digestion process; for example, the in vitro digestion of cowpea (*V. unguiculata*) [22], jackfruit extract [19], and lychee pericarp [23] led to an increase in the total polyphenol content, probably associated with the gradual release of these compounds during the digestive process due to the different composition of the starting food matrices.

With regards to TFC, the trend that has been observed is almost similar to TPC. The metabolism of flavonoids begins with a hydrolysis reaction, which leads to the glycosylation of these compounds [24]. The ability of these aglycones to be absorbed is also influenced by the type of enzyme that carries out the hydrolysis. In the food matrix of honey, there are different kind of enzymes, derived from bees, nectar and pollen, that could modify and further process flavonoids, and consequently alter their accessibility [4]. The flavonoid content in undigested Manuka honey was 48.99 CatEq/Kg honey and decreased by about seven times in the DMH sample (6.88 CatEq/Kg honey). Similar results were found in the work of Guan-Lin Chen et al., that evaluated how the flavonoid content changed after digestion of 23 different types of edible flowers, and a marked decrease was noted in all types of tested sample [25].

TAC was evaluated by FRAP, TEAC and DPPH methods. It must be taken into consideration that, as mentioned in the introduction, inside honey there are other bioactive compounds (vitamins, organic acids, proteins, enzymes), in addition to phenolic compounds, that possess a certain antioxidant activity or interact with phenolic compounds present in the matrix, ensuring that their antioxidant capacity is preserved, despite the chemical and structural changes that occur during the gastrointestinal digestion process [26]. A higher similarity between FRAP and TEAC has been detected, in which the antioxidant capacity of MH and DMH is only decreased by about two times its original amount. The TAC in MH, evaluated with FRAP and TEAC, was 261.73 and 251.89 µmol TEq/100 g honey, respectively, and was lowered in the sample representing the bioaccessible intestinal fraction to values of 119.81 and 114.67, respectively. The DPPH test, as in the studies of O’Sullivan et al. [16] and Seraglio et al. [27], showed a greater decrease in the undigested and the digested sample, suggesting that it is more specific to phenolic compounds than the other two methods used. In fact, the TAC value obtained from the MH (86.479 µmol TEq/100 g honey) decreased by approximately seven times (11.8214 µmol TEq/100 g honey) in DMH as the trend ofTPC and TFC.

#### 3.1.2. Phenolic Profiling by HPLC-ESI-MS/MS

The phenolic compounds, phenolic acids and flavonoids identified in MH and DMH are reported in Table 2.

As shown in the table, phenolic acids appear to be more stable during the gastrointestinal digestion process; in particular, they show that 3,4 dihydroxybenzoic acid maintains approximately the same quantities in the digested sample. As for the other phenolic acids present, such as ferulic acid, *p*-coumaric acid and syringic acid, there was a decrease in the bioaccessible fraction, but salicylic acid’s (SA) intestinal fraction increased from 42.40 μg/kg honey in MH to 56.23 in DMH, as reported in other works [27]. This result could be due to the demethylation processes of methyl salicylate enzymes, widely present in raw honey, and because these phenolic acids could be linked to other molecules such as sugars, that protect them during the digestion process [28]. It is interesting to note that the SA is responsible for the antioxidant activity of honey, as highlighted in a study carried out by Parker et al., which evaluated the antioxidant effect of different compounds present in honey (not only phenolics ones) through ORAC before and after a simulated digestion process, highlighting the synergistic action of this phenolic acid with the sugars present in honey, and with the transformation processes that occur in the digestion process [29].

**Figure 2:**
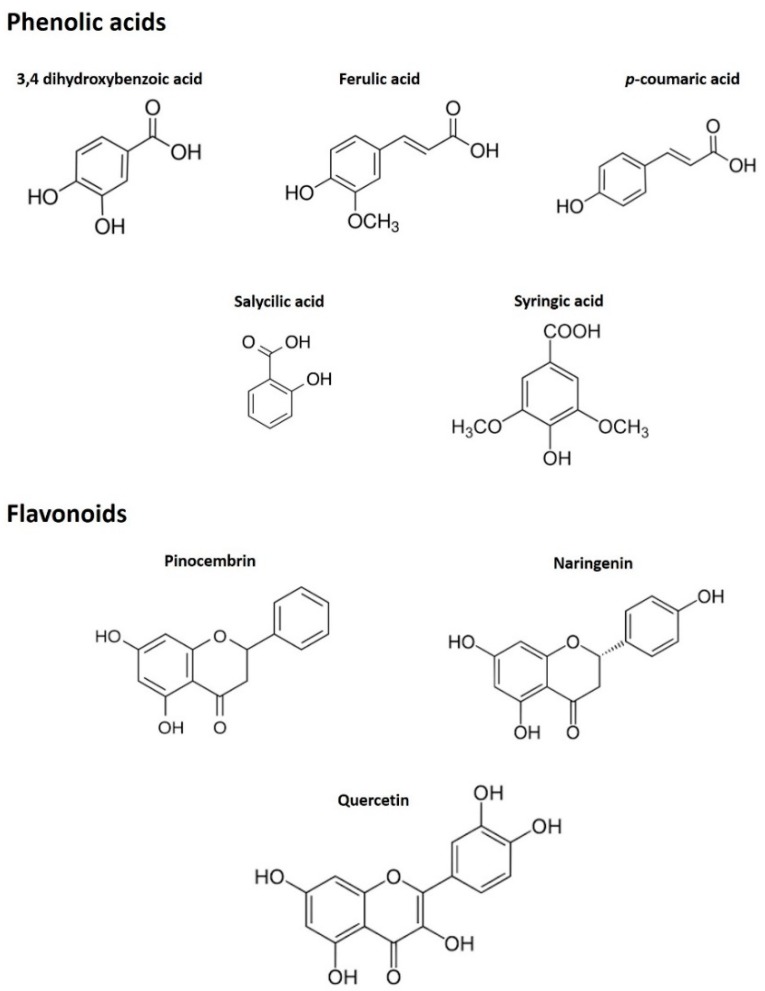
Chemical structures of the principal phenolic compounds detected by HPLC-ESI-MS/MS.

Regarding the evaluation of the concentration of flavonoids in the two types of sample, a drastic reduction was noted. The presence of pinocembrin (102.20 μg/kg honey) was predominant in MH, followed by naringenin and quercetin. Pinocembrin underwent a decrease after the digestive process up to 0.47 μg/kg, while the other compounds were even not detected in the digested sample. A possible explanation for this difference in the stability of the various phenolic compounds could be the higher sensitivity to the pH of the multiring phenolic compounds compared to the monoring ones [30], which could lead to their easier and frequent degradation during the digestion process, in which the pH change is considerable. In fact, observing the chemical structure (Figure 2) of the principal phenolic compounds detected by HPLC-ESI-MS/MS, it can be seen that there is a clear correspondence between the decrease in the quantity of the compound after digestion and the complexity of their structure.

### 3.2. Antiproliferative Effect of MH and DMH on HCT-116 Cells

The antiproliferative effect of MH and DMH samples at concentrations in the range 0–26 mg/mL for 24, 48 and 72 h was assessed in HCT-116 cells using an MTT assay.

**Figure 3:**
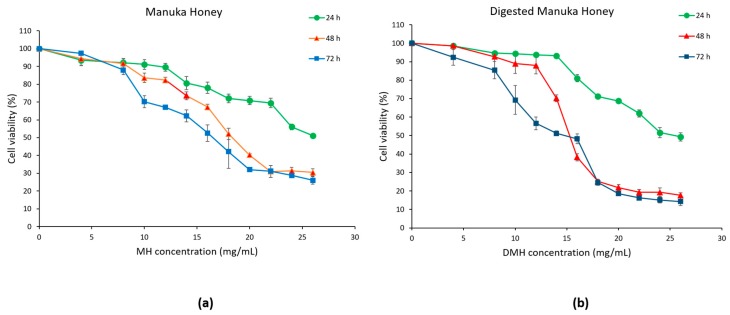
Inhibition of cell proliferation by MH (**a**) and DMH (**b**) in HCT-116 cells. The cells were treated with different concentrations of MH or DMH for 24, 48 and 72 h. Cell viability was measured using MTT and the results were expressed as % of viable cells. All data are expressed as the mean of three independent experiments ± SD.

As shown in Figure 3a,b, cell viability was reduced in a dose- and time-dependent manner in cells treated with MH or DMH compared to the control (untreated cells). In colorectal adenocarcinoma cells, IC_50_ values (concentration required for 50% inhibition of cell growth) of MH were 30.2 mg/mL at 24 h, 19.16 mg/mL at 48 h and 16.97 mg/mL at 72 h.

Comparable results were obtained in our previous study that evaluated the effect of MH on HCT-116, while the IC_50_ value of MH in LoVo, a metastatic cell line, was much higher: values of 62.85 mg/mL were obtained at 24 h, 40.97 mg/mL at 48 h and 22.73 mg/mL at 72 h [31].

Similar results were obtained on another colorectal cancer cell line (HT-29), in which the dose of MH required to reach IC_50_ was 20 mg/mL at 24 h and 10 mg/mL at 72 h [32]. Gelam honey was able to inhibit 50% of cell growth of HT-29 with a dose of 36.2 mg/mL after 72 h [33]. Lower results were obtained with Strawberry tree honey, which showed an IC_50_ of HCT-116 equal to 13.34 mg/mL at 24 h, 9.48 mg/mL at 48 h and 8.76 mg/mL at 72 h [34]. Even more inferior were the results obtained by Imtara et al., which achieved IC_50_ values around 2 mg/mL with different types of honey from Morocco and from Palestine after 72 h of treatment of HCT-116 [35]. These differences are mostly due to the different compositions of the different types of honey.

As regards to the digested sample, a greater, if slight, cytotoxic effect was found on HCT-116. The values of IC_50_ were as follows: 28.36 mg/mL at 24 h, 16.11 mg/mL at 48 h and 14.32 mg/mL at 72 h. As mentioned in the introduction, there are few works that evaluate the biological effect of digested honey compared to the corresponding raw honey. This was done only in the work of O’Sullivan et al., which evaluated the effect of four different types of commercial honey on Caco-2 cells (human epithelial colorectal adenocarcinoma cells), observing that the dose needed to inhibit 50% of cell growth was lower in digested samples (0.5–2 mg/mL) compared to the undigested sample (over 6 mg/mL) [16]. Similar results were also found in the work of Ariza et al., in which the IC_50_ of the HepG2 (human liver carcinoma cells) of the digested samples of strawberries and achenes were lower than the respective raw fruits [36]. Kubow et al. reported that, after the digestion of two different species of purple-fleshed potatoes, their cytotoxicity on Caco-2 cells increased [37]. The higher cytotoxic effect of the digested fraction, despite the lower amount of phenolic compounds, flavonoids and antioxidant capacity, could be due to the production of metabolites with a more antiproliferative effect, but more studies will have to be carried out to understand the real effect of digestion on the production of potentially cytotoxic metabolites from the compounds initially present in the food matrix.

After carefully analyzing the results obtained from the MTT assay, concentrations of 8, 16 and 24 mg/mL, that, in the case of DMH, correspond to 54, 108 and 162 mg/mL of fresh honey, respectively, were chosen for the following experiments. In order to compare the two samples, we decided to use the same concentrations for both. In addition, a 48 h treatment time was chosen. The selected concentrations correspond to approximately 80%, 50% and 20% of cell viability.

### 3.3. Effect of MH and DMH on Intracellular ROS Production

**Figure 4:**
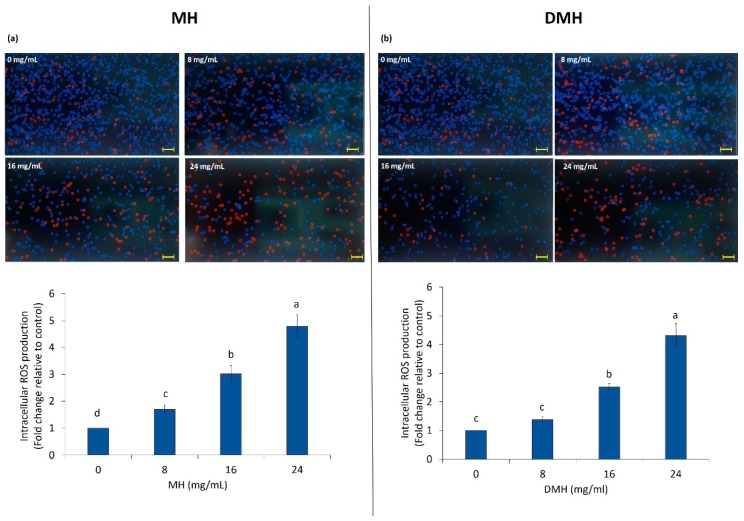
MH (**a**) and DMH (**b**) induce ROS production in HCT-116 cells. The cells were treated with different concentrations (0, 8, 16 and 24 mg/mL) of MH or DMH for 48 h. Intracellular ROS accumulation in HCT-116 was determined by CellROX^®^ Orange assay kit with Tali^®^ Image-Based Cytometer. Values are expressed as the mean of three independent experiment ± SD. The different superscript letters (a–d) in the bars are significantly different (*p* < 0.05). The images are representative of intracellular ROS quantification; red color cells represent ROS-induced cells. Scale bar = 50 μm.

As shown in Figure 4a,b, treatment of HCT-116 significantly increased (*p* < 0.05) the intracellular ROS levels in all concentrations of both samples except for the lower concentration of DMH, where the production of ROS was statistically (*p* < 0.05) similar to the control.

The evaluation of ROS production after MH treatment had already been done in our previous work [38], and the results found are almost similar to those obtained in this work. As regards the comparison between the effect of the two different samples (MH and DMH) on the production of ROS, there is not much difference. MH increased the production of ROS a little more than DMH. With a concentration of 8 mg/mL, the increase in ROS production compared to the control, was 1.7% for MH and 1.3% for DMH treatment; treatment with 16 mg/mL of MH increased the intracellular levels of ROS by 3% and, with the same concentration of DMH, the increase was about 2.5%. The highest concentration used increased ROS by 4.8% and 4.3%, respectively, compared to the control.

The double role of ROS is well known; these compounds have a deleterious effect if produced in physiological conditions, while in pathological conditions, like cancer, these species can help to induce apoptosis, and therefore program cell death [39]. There are several works in which the treatment of colorectal cancer cells with different types of honey caused a similar pattern in the production of intracellular ROS: strawberry tree honey induced a greater accumulation of ROS in both HCT-116 than in the LoVo cells [34] and a commercial honey of Indian origin caused the same increase in HCT-116 and HT-29 [40]. Similar results were found with a multifloral honey and honeydew honey, which managed to increase ROS levels in gastric adenocarcinoma cells in a concentration-dependent manner [41].

Regarding digested samples, very few studies evaluated their biological effects. There are no studies evaluating the effect of digested honey on the intracellular accumulation of ROS, and no study has ever been carried out on the effects of other food matrices on ROS production in cancer cells. The few studies that exist evaluate how the digested sample is able to counteract the accumulation of ROS after chemical stress has been induced (AAPH, H_2_O_2_) [39,42].

### 3.4. Effect of MH and DMH on Apoptosis

**Figure 5:**
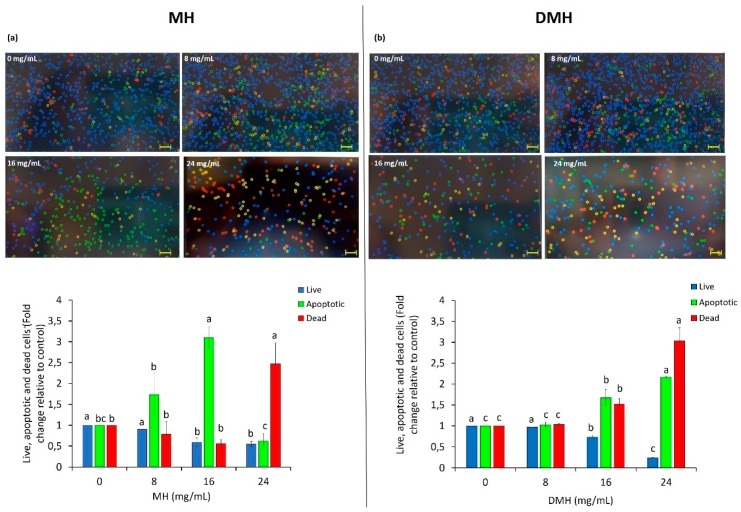
Apoptosis induction by MH (**a**) and DMH (**b**) in HCT-116 cells. The cells were treated with different concentration (0, 8, 16 and 24 mg/mL) of MH or DMH for 48 h. Live, apoptotic and dead cells in HCT-116 were determined by Annexin V Alexa Fluor^®^ 488 and PI staining was done with Tali^®^ Image-Based Cytometer. The images are representative of the effect of MH and DMH with or without treatment: blue corresponds to live cells, green to apoptotic cells and the red or yellow color to dead cells. Values are expressed as the mean of three independent experiment ± SD. The different superscript letters (a–c) in each bar indicate significant difference (*p* < 0.05). Scale bar = 50 μm.

One of the characteristics of cancer cells is their ability to escape the phenomena of cell death such as apoptosis. One of the targets of anticancer therapies is to induce apoptosis in cancer cells [43]. Our results showed that both MH and DMH were able to increase the number of cells in apoptosis, with different behavior. The most significant increase in the number of apoptotic cells was noted with MH treatment at a concentration of 16 mg/mL (Figure 5a); the percentage of apoptotic cells increased by 3.10 fold compared to non-treated cells, a result was similar to that obtained by Afrin et al., who found a 3.38-fold after treating HCT-116 with a concentration of MH equal to 15 mg/mL [31]. It is interesting to note that with the increased concentration of MH (24 mg/mL), the number of apoptotic cells decreased, and the number of dead ones significantly increased (2.47-fold higher than in the control). The treatment of cells derived from colorectal adenocarcinoma with the corresponding digested sample (Figure 5b) had a more linear trend: the increase in apoptotic and dead cells gradually increased with the scalar concentrations of DMH used. With 8 mg/mL of DMH, no significant statistical differences (*p* < 0.05) was noted with respect to the control, while a significant increase in the number of apoptotic cells was reached with the concentration corresponding to the IC_50_ value (the number of apoptotic cells was 1.67-fold more than in the untreated cells), an increase that was even greater with the highest concentration (24 mg/mL), which increased the number of cells in apoptosis 2.16-fold compared to the control. This difference in the effect of the two types of sample on the phenomenon of apoptosis could derive from the single effect that bioactive compounds, in particular phenolic compounds, present in honey, have on the genes that regulate the process of cell death. In fact, as shown by the HPLC characterization, there is a great difference in the composition and quantity of phenolic compounds, and this could change their way of acting and interacting with each other, causing this event. 

Meanwhile, there are studies that evaluated the apoptotic effect of some single compounds also found in MH and DMH, and pinocembrin, the flavonoid mostly present in MH with the greatest loss after the process of in vitro digestion, exerts a particularly elevated proapoptotic effect on HCT-116, promoting Bax translocation [44], while in another study it was observed, for example, that SA acts by increasing the apoptosis rate of HCT-116 cells by repressing survivin expression and increasing that of c-PARP [45]. Further studies at the molecular level are needed to better understand this difference.

Honey has been extensively investigated for its effect on apoptosis in different types of cancer, with different proposed mechanisms of action. On a promyelocytic leukemia cell line (HL-60), it was observed that different types of honey (heater, rosemary and multifloral) were able to significantly increase the number of apoptotic cells in an ROS-independent manner [46]. A similar effect has been seen in renal carcinoma cells treated with Iranian multifloral honey [47]. In breast cancer cells (MDA-MB-231 and MCF-7), a significant increase in apoptotic cells was noted after treatment with Tualang honey through the activation of caspases 3, 7 and 9 [48]. A proapoptotic effect, due to the increase in caspases, especially caspase 8 and 9, as well as p53 and poly ADP ribose polymerase (PARP), has been seen in our previous work [31], where, as previously mentioned, MH had a proapoptotic effect on HCT-116 and LoVo cell lines. With regard to colorectal cancer, the effect of MH on apoptosis was also confirmed on other cell lines (HCT-15 and HT29) with an ROS-dependent mechanism and with an increase in p53, caspase3 and Bax and a decrease in PARP [40]; similar results, with the activation of the same molecular patterns, have been seen in a human hepatic carcinoma cell line (HepG2) and human bladder carcinoma cells (5637) treated with Astragalus honey [49].

As regards as the effect of digested samples on apoptosis, as with the evaluation of the biological effects considered previously, there is a small amount of studies. In the only two that evaluate the effect of digested samples on apoptosis, a comparison with the undigested sample is not carried out; despite this, an increase in the number of apoptotic cells was noted in colorectal adenocarcinoma (HT-29 and HCT-116) after treatment with digested tomatoes through the regulation of cyclin D1, Bcl-2 and Bcl-xL [50]. An increased rate of apoptosis has also been noted in Caco-2 cells after treatment with fruit beverages subjected to in vitro gastrointestinal digestion [51].

### 3.5. MH and DMH Induce Cell Cycle Arrest

**Figure 6:**
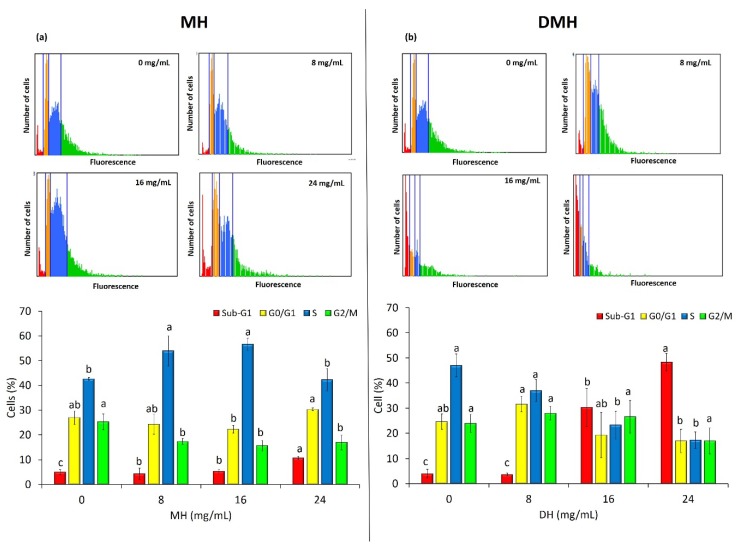
MH (**a**) and DMH (**b**) induce cell cycle arrest in HCT-116 cells. The cells were treated with different concentrations (0, 8, 16 and 24 mg/mL) of MH or DMH for 48 h. The percentages of cells in Sub-G1 (red; apoptotic cells), G0/G1 (yellow), S (blue) and G2/M (green) were calculated by Tali^®^ Image-Based Cytometer. The fluorescence images are representative of the effect of MH and DMH in the cell cycle of HCT-116. Values are expressed as the mean of three independent experiment ± SD. The different superscript letters (a–c) in each bar indicate significant difference (*p* < 0.05).

The development and progression of cancer are closely associated with disorders in the regulation of cell cycle, so the use of substances that are able to effect the regulation of this process is very important and could represent a good strategy for chemoprevention to counteract tumor growth and proliferation [52]. Since an antiproliferative effect on HCT-116 was noted, exerted both by MH and DMH, their effect on the cell cycle was evaluated using the Tali^®^ Image-Based Cytometer. As shown in Figure 6, the two different types of samples behaved differently in regulating the cell cycle of HCT-116. The MH (Figure 6a), at a concentration of 16 mg/mL, increased the number of cells in the S phase by about 14% compared to the control, while, at the same concentration, we noted a significant decrease (*p* < 0.05) in the number of cells in the G0/G1 phase (4.67% less than the control) and in the G2/M phase (9.67% less than the control). It is therefore possible to state that MH is able to stop cells in the S phase of the cell cycle. Similar results have been obtained in our previous work in HCT-116 [30], and also with Tualang honey, which was able to stop the cell cycle of breast cancer cells (MDA-MB-231) in S phase [48].

As regards the regulation of the cell cycle by DMH, a statistically significant increase (*p* < 0.05) can be noted in the number of cells present in the Sub-G1 phase, including both apoptotic and oncotic cells: the percentage of cells in this phase was, respectively, 26% and 44% more than control with treatment at 16 and 24 mg/mL. On the other hand, there was a significant decrease (*p* < 0.05) in the number of cells in S phase, up to a decrease of 29.67% with the highest treatment compared to the control, and in the number of cells in the G0/G1 phase (7%). Even for this type of analysis, it is difficult to make a comparison with other works: only Palozza et al. evaluated the effect of in vitro digested tomatoes on HT-29 and HCT-116 cells, noting a block in the progression of the cell cycle in the G0/G1 phase [50].

This difference in the effects of the two different treatments in the regulation of the cell cycle is probably due to the different contents of the bioactive compounds, in particular phenolic compounds. Some papers that have investigated the effect on the cell cycle of some of the single compounds identified with HPLC. As regards flavonoids (which are almost completely absent in the digested fraction), pinocembrin was found to block the cell cycle of human prostatic cancer cells (LNCaP) in the S phase [53] and the same effect was observed in hepatocellular carcinoma (HepG2) [54]. Another flavonoid found only in honey not subjected to digestion, quercetin, has shown a similar effect, blocking the CSCs derived from colorectal carcinoma (HT-29) in the S phase [55], and also in three leukemic cell lines (CEM, K562, Nalm6) and in two breast cancer cell lines (T47D and EAC) [56].

Of the phenolic acids, which the digested fraction was rich in, we noted, in line with our results, that 3,4 dihydroxybenzoic acid and its derivatives increased the number of cells in the sub-phase G1 of HCT-116 [57]. Ferulic acid is also able to induce cell cycle arrest in the same phase in cervical cancer cells (Hela and Caski cells) [58].

### 3.6. MH and DMH Reduced Colony Formation Ability of HCT-116

**Figure 7:**
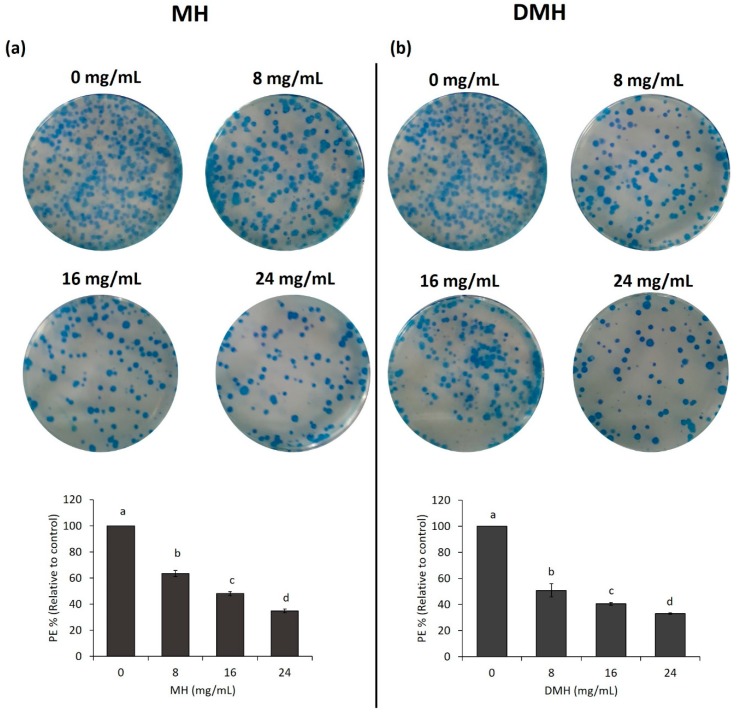
MH (**a**) and DMH (**b**) inhibits colony formation ability by MH and DMH in HCT-116. Cells were treated with different concentrations (0, 8, 16 and 24 mg/mL) of MH or DMH for 48 h. After treatment the cells were seeded at a density of 1000 cells/well for 12 days. The formed colonies were fixed with 70% ethanol and stained with methylene blue for count colonies. The results are expressed as a % of plating efficiency (PE) relative to control. The values are expressed as the mean of three independent experiments ± SD. The different superscript letters (a–d) in each bar indicate significant difference (*p* < 0.05).

As shown in Figure 7a,b, both MH and DMH were able to inhibit the formation of colonies in HCT-116 cells; in particular, DMH had a greater inhibitory capacity in its lower concentration (8 mg/mL) compared to the same concentration of MH, decreasing the colonigenic potential of cells by 51%, compared to 39% with MH. In the other two concentrations tested (16 and 24 mg/mL), the decrease in the number of formed colonies for both treatments was almost similar, up to a decrease of about 65% compared to the control. This activity of MH has also been confirmed in another colon cancer cell line (LoVo) where a concentration of 50 mg/mL reduced colony formation by up to 80% [37], and in one breast cancer line (MDA-MB-231), where the higher concentration of MH decreased this value by up to 60% [59]. Similar results were obtained with propolis, which decreased the colonigenic capacity of two cell lines of glioblastoma (U251, U343) [60].

## 4. Conclusions

In the present study, the effect of in vitro gastrointestinal digestion of Manuka honey was investigated, evaluating how the content of phenolic compounds and antioxidant activity changed, and studying whether this modification could affect the biological activities of honey in a human colon adenocarcinoma cell line (HCT-116). It was observed that the content of phenolic compounds drastically changed between digested and undigested honey, especially with regard to flavonoids, which are almost undetectable in the digested fraction, probably because of their more complex structure and their instability due to pH changes occurring during the gastrointestinal digestion process. Although change in the content of phenolic compounds was evident, the antioxidant capacity decreased much less, because in honey there are also other bioactive compounds that are able to exert this function. Regarding the anti-tumor activity against HCT-116, both treatments led to similar results regarding intracellular ROS production and inhibition of colony formation. However, some differences have been observed in the induction of apoptosis and the regulation of the cell cycle, resulting in a block of cells in two different phases (S for MH and Sub G1 for DMH). These differences are probably due to the different effects that the single compounds have on the regulation of apoptosis and cell cycle. These results increase knowledge of the effect of gastrointestinal digestion on the biological effect of honey against colorectal cancer; deeper molecular investigation are needed to better understand the real mechanism behind the demonstrated biological effect.

## Figures and Tables

**Table 1:** Phytochemical composition and antioxidant capacity of MH and DMH.

Samples	TPC (mg GAEq/g Honey)	TFC (mg CatEq/Kg Honey)	TAC		
			FRAP (µmol FEq/100 g Honey)	TEAC (µmol TEq/100 g Honey)	DPPH (µmol TEq/100 g Honey
**MH**	1.27 ± 0.08 ^a^	48.99 ± 1.44 ^a^	261.73 ± 3.65 ^a^	251.89 ± 4.74 ^a^	86.479 ± 2.60 ^a^
**DMH**	0.203 ± 0.01 ^b^	6.49 ± 0.96 ^b^	119.81 ± 0.55 ^b^	114.67 ± 4.66 ^b^	11.821 ± 2.25 ^b^

MH = Manuka honey; DMH = digested Manuka honey; TPC = Total phenolic content; GAEq = Gallic acid equivalent; TFC = total flavonoids content; TAC = total antioxidant capacity; FRAP = ferric ion reducing antioxidant power; FEq = ferrous equivalents; TEAC = trolox equivalents antioxidant capacity; TEq =Trolox equivalents; DPPH = 2,2-diphenyl-1-picrylhydrazyl. Results are expressed by the mean ± SD (*n* = 3). Different letters (a, b) in the same column indicate significant differences (*p* < 0.05).

**Table 2:** Quantification ion, retention time and concentration of phenolic compounds in Manuka honey and in the fraction obtained after in vitro digestion (μg/kg honey).

Compounds	Quantification Ion	Retention Time (min)	Concentration of Phenolic Compounds	
			Manuka Honey	Digested Manuka Honey
***Phenolic acid***				
3,4 dihydroxybenzoic acid	109	14.29	17.10 ± 1.01	15.73 ± 1.36
Ferulic acid	89	13.77	3.00 ± 0.18	0.60 ± 0.10
*p*-coumaric acid	119	13.10	11.20 ± 0.66	1.70 ± 0.20
Salicylic acid	93	14.43	42.40 ± 2.50	56.23 ± 7.91
Syringic acid	155	12.42	1.70 ± 0.10	0.43 ± 0.15
*Σ phenolic acids*			*75*	*75*
***Flavonoids***				
Pinocembrin	153	16.36	102.20 ± 6.04	0.47 ± 0.06
Naringenin	151	15.25	6.10 ± 0.36	n.d.
Quercetin	150	14.50	7.50 ± 0.44	n.d
*Σ flavonoids*			*116*	*1*
***Total phenolics***			***191***	**76**

n.d. not detected; Results are expressed by mean ± SD (*n* = 3).

## References

[1] Baena R., Salinas P. (2015). Diet and colorectal cancer. Maturitas.

[2] Thanikachalam C., Khan G. (2019). Colorectal Cancer and Nutrition. Nutrients.

[3] Battino M., Forbes-Hernández T.Y., Gasparrini M., Afrin S., Cianciosi D., Zhang J., Manna P.P., Reboredo-Rodríguez P., Varela Lopez A., Quiles J.L. (2019). Relevance of functional foods in the Mediterranean diet: The role of olive oil, berries and honey in the prevention of cancer and cardiovascular diseases. Crit. Rev. Food Sci. Nutr. Chem..

[4] Cianciosi D., Forbes-Hernández T.Y., Afrin S., Gasparrini M., Reboredo-Rodriguez P., Manna P.P., Zhang J., Bravo L., Martínez F.S., Agudo T.P. (2018). Phenolic Compounds in Honey and Their Associated Health Benefits: A Review. Molecules.

[5] Teng H., Chen L. (2019). Polyphenols and bioavailability: An update. Crit. Rev. Food Sci. Nutr..

[6] Mandalari G., Vardakou M., Faulks R., Bisignano C., Martorana M., Smeriglio A., Trombetta D. (2016). Food Matrix Effects of Polyphenol Bioaccessibility from Almond Skin during Simulated Human Digestion. Nutrients.

[7] Bohn T., Carriere F., Day L., Deglaire A., Egger L., Freitas D., Golding M., Le Feunteun S., Macierzanka A., Menard O. (2018). Correlation between in vitro and in vivo data on food digestion. What can we predict with static in vitro digestion models?. Crit. Rev. Food Sci. Nutr..

[8] Gil-Izquierdo A., Zafrilla P., Tomás-Barberán F.A. (2002). An in vitro method to simulate phenolic compound release from the food matrix in the gastrointestinal tract. Eur. Food Res. Technol..

[9] Singleton V.L., Orthofer R., Lamuela-Raventós R.M. (1999). Analysis of total phenols and other oxidation substrates and antioxidants by means of folin-ciocalteu reagent. Methods Enzymol..

[10] Ariza M.T., Reboredo-Rodríguez P., Mazzoni L., Forbes-Hernández T.Y., Giampieri F., Afrin S., Gasparrini M., Soria C., Martínez-Ferri E., Battino M. (2016). Strawberry Achenes Are an Important Source of Bioactive Compounds for Human Health. Int. J. Mol. Sci..

[11] Deighton N., Brennan R., Finn C., Davies H.V. (2000). Antioxidant properties of domesticated and wild Rubus species. J. Sci. Food Agric..

[12] Re R., Pellegrini N., Proteggente A., Pannala A., Yang M., Rice-Evans C. (1999). Antioxidant activity applying an improved ABTS radical cation decolorization assay. Free Radic. Biol. Med..

[13] Kumaran A., Karunakaran R.J. (2007). In vitro antioxidant activities of methanol extracts of five Phyllanthus species from India. Food Sci. Techol..

[14] Seraglio S.K.T., Valese A.C., Daguer H., Bergamo G., Azevedo M.S., Gonzaga L.V., Fett R., Costa A.C.O. (2016). Development and validation of a LC-ESI-MS/MS method for the determination of phenolic compounds in honeydew honeys with the diluted-and-shoot approach. Food Res. Int..

[15] Waghela B.N., Sharma A., Dhumale S., Pandey S.M., Pathak C. (2015). Curcumin conjugated with PLGA potentiates sustainability, anti-proliferative activity and apoptosis in human colon carcinoma cells. PLoS ONE.

[16] O’Sullivan A.M., O’Callaghan Y.C., O’Connor T.P., O’Brien N.M. (2013). Comparison of the Antioxidant Activity of Commercial Honeys, Before and After In-Vitro Digestion. Pol. J. Food Nutr. Sci..

[17] Jara-Palacios J.M., Gonçalves S., Hernanz D., Heredia F.J., Romano A. (2018). Effects of in vitro gastrointestinal digestion on phenolic compounds and antioxidant activity of different white winemaking byproducts extracts. Food Res. Int..

[18] Fawole O.A., Opara U.L. (2016). Stability of total phenolic concentration and antioxidant capacity of extracts from pomegranate co-products subjected to in vitro digestion. BMC Complement. Altern. Med..

[19] Pavan V., Soriano Sancho A.R., Pastore G.M. (2014). The effect of in vitro digestion on the antioxidant activity of fruit extracts (*Carica papaya*, *Artocarpus heterophillus* and *Annona marcgravii*). Food Sci. Technol..

[20] Martínez-Las Heras R., Pinazo A., Heredia A., Andrés A. (2017). Evaluation studies of persimmon plant (*Diospyros kaki*) for physiological benefits and bioaccessibility of antioxidants by in vitro simulated gastrointestinal digestion. Food Chem..

[21] Tagliazucchi D., Verzelloni E., Bertolini D., Conte A. (2010). In vitro bio-accessibility and antioxidant activity of grape polyphenols. Food Chem..

[22] Mtolo M., Gerrano A., Mellem J. (2017). Effect of simulated gastrointestinal digestion on the phenolic compound content and in vitro antioxidant capacity of processed Cowpea (*V. unguiculata*) cultivars. CyTA J. Food.

[23] Zeng Q., Xu Z., Dai M., Cao X., Xiong X., He S., Yuan Y., Zhang M., Dong L., Zhang R. (2019). Effects of simulated digestion on the phenolic composition and antioxidant activity of different cultivars of lychee pericarp. BMC Chem..

[24] Spencer J.P., Chowrimootoo G., Choudhury R., Debnam E.S., Srai S.K., Rice-Evans C. (1999). The small intestine can both absorb and glucuronidate luminal flavonoids. FEBS Lett..

[25] Chen G.-L., Chen D.-C., Xie Y.-Q., Chen F., Zhao Y.-Y., Luo C.-X., Gao Y.Q. (2015). Total phenolic, flavonoid and antioxidant activity of 23 edible flowers subjected to in vitro digestion. J. Funct. Foods.

[26] Helal A., Tagliazucchi D., Verzelloni E., Conte A. (2014). Bioaccessibility of polyphenols and cinnamaldehyde in cinnamon beverages subjected to in vitro gastro-pancreatic digestion. J. Funct. Foods.

[27] Seraglio S.K.T., Valese A.C., Daguer H., Bergamo G., Azevedo M.S., Nehring P., Gonzaga L.V., Fett R., Costa A.C.O. (2017). Effect of in vitro gastrointestinal digestion on the bioaccessibility of phenolic compounds, minerals, and antioxidant capacity of Mimosa scabrella Bentham honeydew honeys. Food Res. Int..

[28] Costa J.R., Amorim M., Vilas-Boas A., Tonon R.V., Cabral L.M.C., Pastrana L., Pintado M. (2019). Impact of in vitro gastrointestinal digestion on the chemical composition, bioactive properties, and cytotoxicity of *Vitis vinifera* L. cv. Syrah grape pomace extract. Food Funct..

[29] Parker T.L., Miller S.A., Myers L.E., Miguez F.E., Engeseth N.J. (2010). Evaluation of synergistic antioxidant potential of complex mixtures using oxygen radical absorbance capacity (ORAC) and electron paramagnetic resonance (EPR). J. Agric. Food Chem..

[30] Friedman M., Jürgens H.S. (2000). Effect of pH on the stability of plant phenolic compounds. J. Agric. Food Chem..

[31] Afrin S., Giampieri F., Gasparrini M., Forbes-Hernández T.Y., Cianciosi D., Reboredo-Rodriguez P., Amici A., Quiles J.L., Battino M. (2018). The inhibitory effect of Manuka honey on human colon cancer HCT-116 and LoVo cell growth. Part 1: The suppression of cell proliferation, promotion of apoptosis and arrest of the cell cycle. Food Funct..

[32] Fernandez-Cabezudo M.J., El-Kharrag R., Torab F., Bashir G., George J.A., El-Taji H., Al-Ramadi B.K. (2013). Intravenous administration of manuka honey inhibits tumor growth and improves host survival when used in combination with chemotherapy in a melanoma mouse model. PLoS ONE.

[33] Hashim F., Ismail W.I., Ali A.M. (2019). Combinatorial Cytotoxic Effects of Gelam Honey and 5-Fluorouracil against Human Adenocarcinoma Colon Cancer HT-29 Cells In Vitro. Int. J. Cell Biol..

[34] Afrin S., Forbes-Hernandez T.Y., Gasparrini M., Bompadre S., Quiles J.L., Sanna G., Spano N. (2017). Strawberry-Tree Honey Induces Growth Inhibition of Human Colon Cancer Cells and Increases ROS Generation: A Comparison with Manuka Honey. Int. J. Mol. Sci..

[35] Imtara H., Kmail A., Touzani S., Khader M., Hamarshi H., Saad B., Lyoussi B. (2019). Chemical Analysis and Cytotoxic and Cytostatic Effects of Twelve Honey Samples Collected from Different Regions in Morocco and Palestine. Evid. Based Complement. Altern. Med..

[36] Ariza M.T., Forbes-Hernández T.Y., Reboredo-Rodríguez P., Afrin S., Gasparrini M., Cervantes L., Soria C., Martínez-Ferri E., Battino M., Giampieri F. (2018). Strawberry and Achenes Hydroalcoholic Extracts and Their Digested Fractions Efficiently Counteract the AAPH-Induced Oxidative Damage in HepG2 Cells. Int. J. Mol. Sci..

[37] Kubow S., Iskandar M.M., Melgar-Bermudez E., Sleno L., Sabally K., Azadi B., How E., Prakash S., Burgos G., Felde T.Z. (2017). Effects of Simulated Human Gastrointestinal Digestion of Two Purple-Fleshed Potato Cultivars on Anthocyanin Composition and Cytotoxicity in Colonic Cancer and Non-Tumorigenic Cells. Nutrients.

[38] Afrin S., Giampieri F., Gasparrini M., Forbes-Hernández T.Y., Cianciosi D., Reboredo-Rodriguez P., Manna P.P., Zhang J., Quiles J.L., Battino M. (2018). The inhibitory effect of Manuka honey on human colon cancer HCT-116 and LoVo cell growth. Part 2: Induction of oxidative stress, alteration of mitochondrial respiration and glycolysis, and suppression of metastatic ability. Food Funct..

[39] Circu M.L., Aw T.Y. (2010). Reactive oxygen species, cellular redox systems, and apoptosis. Free Radic. Biol. Med..

[40] Jaganathan S.K., Mandal M. (2010). Involvement of non-protein thiols, mitochondrial dysfunction, reactive oxygen species and p53 in honey-induced apoptosis. Investig. New Drugs.

[41] Kocyigit A., Aydogdu G., Balkan E., Yenigun V.B., Guler E., Bulut H., Koktasoglu F., Gören A.C., Atayoglu A.T. (2019). Quercus pyrenaica Honeydew Honey with High Phenolic Contents Cause DNA Damage, Apoptosis, and Cell Death Through Generation of Reactive Oxygen Species in Gastric Adenocarcinoma Cells. Integr. Cancer Ther..

[42] Chen W., Su H., Xu Y., Jin C. (2017). In vitro gastrointestinal digestion promotes the protective effect of blackberry extract against acrylamide-induced oxidative stress. Sci. Rep..

[43] Pfeffer C.M., Singh A.T.K. (2018). Apoptosis: A Target for Anticancer Therapy. Int. J. Mol. Sci..

[44] Kumar M.A., Nair M., Hema P.S., Mohan J., Santhoshkumar T.R. (2007). Pinocembrin triggers Bax-dependent mitochondrial apoptosis in colon cancer cells. Mol. Carcinog..

[45] Pathi S., Jutooru I., Chadalapaka G., Nair V., Lee S.O., Safe S. (2012). Aspirin inhibits colon cancer cell and tumor growth and downregulates specificity protein (Sp) transcription factors. PLoS ONE.

[46] Morales P., Haza A.I. (2013). Antiproliferative and apoptotic effects of spanish honeys. Pharmacogn. Mag..

[47] Samarghandian S., Afshari J.T., Davoodi S. (2011). Honey induces apoptosis in renal cell carcinoma. Pharmacogn. Mag..

[48] Fauzi A.N., Norazmi M.N., Yaacob N.S. (2011). Tualang honey induces apoptosis and disrupts the mitochondrial membrane potential of human breast and cervical cancer cell lines. Food Chem. Toxicol..

[49] Sadeghi-Aliabadi H., Hamzeh J., Mirian M. (2015). Investigation of Astragalus honey and propolis extract’s cytotoxic effect on two human cancer cell lines and their oncogen and proapoptotic gene expression profiles. Adv. Biomed. Res..

[50] Palozza P., Serini S., Boninsegna A., Bellovino D., Lucarini M., Monastra G., Gaetani S. (2007). The growth-inhibitory effects of tomatoes digested in vitro in colon adenocarcinoma cells occur through down regulation of cyclin D1, Bcl-2 and Bcl-xL. Br. J. Nutr..

[51] Cilla A., Gonzàles-Sarrìas A., Tomàs-Barberàn F.A., Espìn J.C., Barberà R. (2009). Availability of polyphenols in fruit beverages subjected to in vitro gastrointestinal digestion and their effects on proliferation, cell-cycle and apoptosis in human colon cancer Caco-2 cells. Food Chem..

[52] Meeran S.Y., Katiyar S.K. (2008). Cell cycle control as a basis for cancer chemoprevention through dietary agents. Front. Biosci..

[53] Chen Z., Rasul A., Zhao C., Millimouno F.M., Tsuji I., Yamamura T., Iqbal R., Malhi M. (2013). Antiproliferative and apoptotic effects of pinocembrin in human prostate cancer cells. Bangladesh J. Pharmacol..

[54] Kumar N., Biswas S., Hosur Shrungeswara A., Basu Mallik S., Hipolith Viji M., Elizabeth Mathew J., Mathew J., Nandakumar K., Lobo R. (2017). Pinocembrin enriched fraction of *Elytranthe parasitica* (L.) Danser induces apoptosis in HCT 116 colorectal cancer cells. J. Infect. Chemother..

[55] Atashpour S., Fouladdel S., Komeili Movahhed T., Barzegar E., Hossein Ghahremani M., Nasser Ostad S. (2015). Quercetin induces cell cycle arrest and apoptosis in CD133+ cancer stem cells of human colorectal HT29 cancer cell line and enhances anticancer effects of doxorubicin. Iran. J. Basic Med. Sci..

[56] Srivastava S., Somasagara R.R., Hegde M., Nishana M., Tadi S.K., Srivastava M., Choudhary B., Raghavan S.C. (2016). Quercetin, a Natural Flavonoid Interacts with DNA, Arrests Cell Cycle and Causes Tumor Regression by Activating Mitochondrial Pathway of Apoptosis. Sci. Rep..

[57] Anantharaju P.G., Reddy B.D., Padukudru M.A., Kumari Chitturi C.M., Vimalambike M.G., Madhunapantula S.V. (2017). Naturally occurring benzoic acid derivatives retard cancer cell growth by inhibiting histone deacetylases (HDAC). Cancer Biol. Ther..

[58] Gao J., Yu H., Guo W., Kong Y., Gu I., Li Q., Yang S., Zhang Y. (2018). The anticancer effects of ferulic acid is associated with induction of cell cycle arrest and autophagy in cervical cancer cells. Cancer Cell. Int..

[59] Aryappalli P., Al-Qubaisi S.S., Attoub S., George J.A., Arafat K., Ramadi K.B., Mohamed Y.A., Al-Dhaheri M.M., Al-Sbiei A., Fernandez-Cabezudo M.J. (2017). The IL-6/STAT3 Signaling Pathway Is an Early Target of Manuka Honey-Induced Suppression of Human Breast Cancer Cells. Front. Oncol..

[60] Borges K.S., Brassesco M.S., Scrideli C.A., Soares A.E., Tone L.G. (2011). Antiproliferative effects of Tubi-bee propolis in glioblastoma cell lines. Genet. Mol. Biol..

